# Subcorneal Pustular Dermatosis in Childhood: A Case Report and Review of the Literature

**DOI:** 10.1155/2013/424797

**Published:** 2013-01-09

**Authors:** Massimiliano Scalvenzi, Franco Palmisano, Maria Carmela Annunziata, Ernesto Mezza, Immacolata Cozzolino, Claudia Costa

**Affiliations:** ^1^Department of Dermatology, Federico II University, Via Pansini 5, 80131 Naples, Italy; ^2^Department of Biomorphological and Functional Sciences, Federico II University, 80131 Naples, Italy

## Abstract

Subcorneal pustular dermatosis (SCPD, also known as Sneddon-Wilkinson disease) is a rare, benign, chronic, sterile pustular eruption which usually develops in middle-age or elderly women; it is rarely seen in childhood and adolescence. The primary lesions are pea-sized pustules classically described as half-pustular, half-clear flaccid blisters. Histologically the most important feature is a subcorneal accumulation of neutrophils with the absence of spongiosis or acantholysis, although acantholysis may be reported in older lesions. In this paper we present the case of a 7-year-old boy diagnosed with SCPD based on the characteristic clinical and histological features. Dapsone has been successfully used in the treatment of the disease.

## 1. Introduction

Subcorneal pustular dermatosis (SCPD, Sneddon-Wilkinson disease) is a rare chronic, relapsing, pustular eruption that was first described by Sneddon and Wilkinson in [1]. It has been reported more frequently in women in the age group 40–50 years, however this disease can occur more rarely during the childhood [2]. Patients characteristically have a history of a relapsing symmetrical sterile pustular eruption involving the trunk, intertriginous areas, and flexor aspects of the limbs. The face, palms, soles, and mucous membranes are usually spared. The primary lesion is a small pustule arising on normal skin or slightly erythematous base. The pustules classically are described as half-pustular, half-clear fluid blisters that coalesce to form annular or serpiginous patterns. They are flaccid and rupture easily, resulting in superficial scaling, crusting, and faint hyperpigmentation. The condition is benign unless associated with underlying malignancy, which worsens the prognosis [3].

Its exact pathophysiology is unknown and its exact nosological classification is still controversial. The salient histological feature is a subcorneal accumulation of neutrophils with the absence of spongiosis or acantholysis.

Therapeutically, Dapsone is the first-line treatment in SPD [4, 5], and other treatment options are etretinate, acitretin, PUVA, narrow-band (TL-1) UVB phototherapy, and colchicine [6, 7].

## 2. Case Report

A 7-year-old boy was admitted to our clinic with a 3-week-old itchy eruption located on the trunk, on the limbs, and on the face. He had a history of atopic dermatitis, while his familiar anamnesis was negligible. A complete blood count and the studies of serum biochemistry showed normal results; moreover serum protein electrophoresis had negative results. The lesions initially developed on the trunk and upper extremities, then they progressed up to involve almost the whole body surface. The palms, soles, and mucous membrane were spared, and no lymphadenopathy or hepato-splenomegaly was present. There were no abnormalities of the nails and tongue.

**Figure 1:**
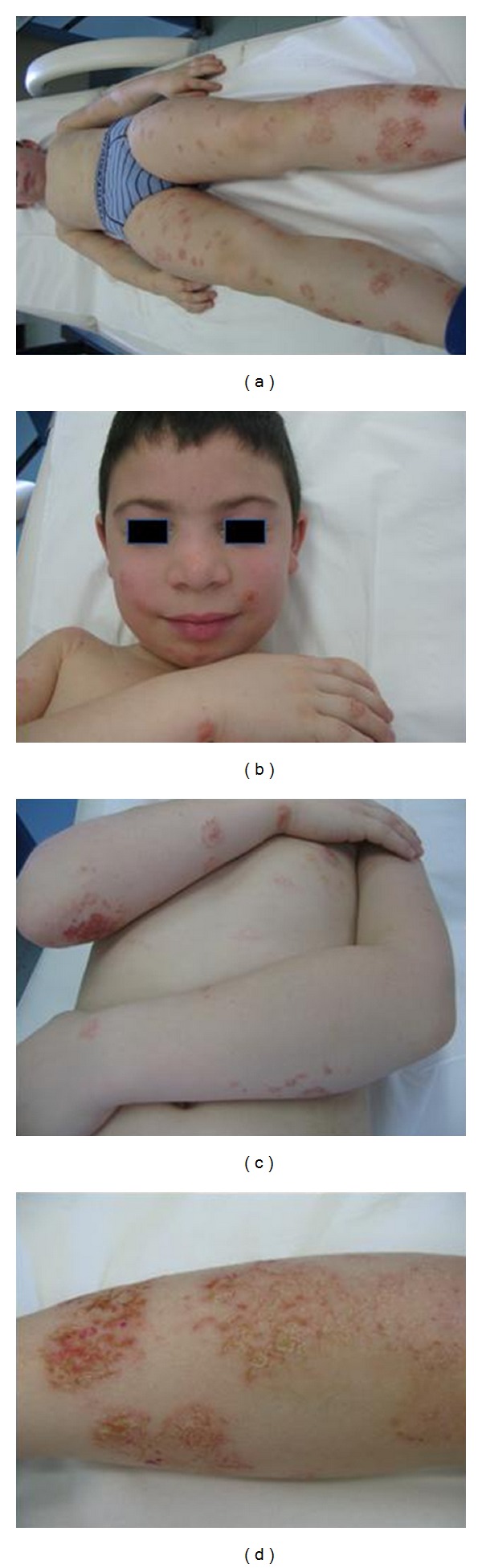
Multiple-grouped flaccid pustules varying in size from 2 to 10 mm that tended to coalesce to form annular, circinate, or serpiginous pattern and superficial crusts on the normal or mildly erythematous skin, of the face, trunk, and extremities.

The dermatologic examination revealed multiple-grouped flaccid pustules varying in size from 2 to 10 mm that tended to coalesce to form annular, circinate, or serpiginous pattern and superficial crusts on the normal or mildly erythematous skin, of the face, trunk, and extremities (Figure 1). Healed lesions presented residual hyperpigmentation and new lesions in the periphery.

Taking into consideration a suspected diagnosis of SCPD, the patient was treated with oral antihistamines and with a topic solution of eosin (2%); moreover an incisional biopsy of a lesion on the sternal region was carried out.

**Figure 2:**
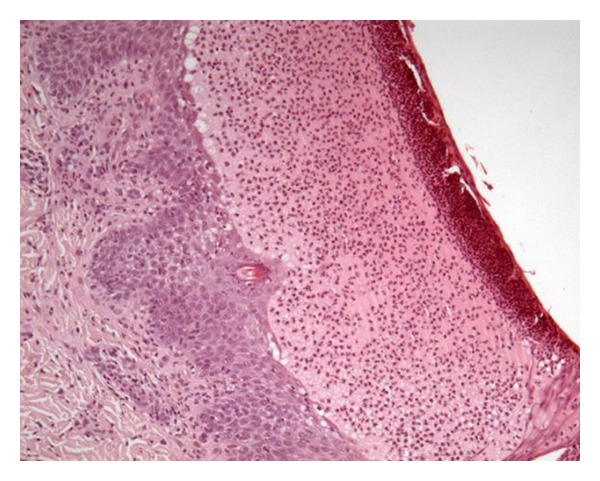
Histological examination: subcorneal pustule immediately below the stratum corneum containing mainly neutrophils; the underlying epidermis show slight intercellular edema (HE ×20).

**Figure 3:**
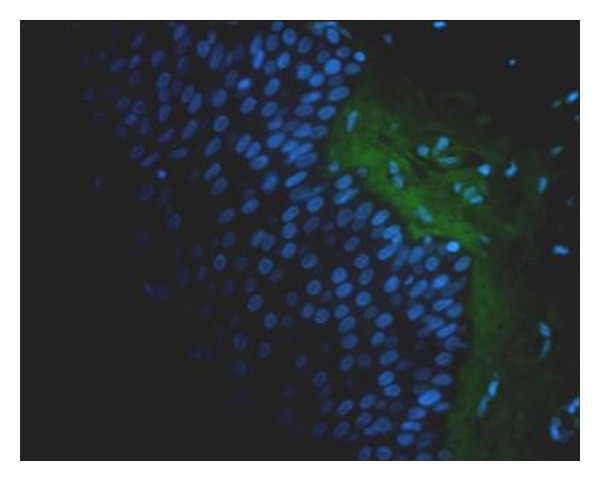
Immunofluorescence. IgA-FITC 40X. The figure shows no antibody reactivity in epithelial cells and dermal-epithelial junction (basement membrane).

Histopathology demonstrated a subcorneal vesiculo-bullous dermatitis (Figure 2); the pustule is located immediately below the stratum corneum and contains mainly neutrophils with few eosinophils. The underlying epidermis to the pustule show slight intercellular edema. In the dermis, superficial blood vessels are surrounded by a nonspecific mixed inflammatory cell infiltrate consisting of neutrophils and mononuclear cells. Direct immunofluorescence studies are negative for immunoglobulin A (IgA) intercellular accumulation (Figure 3). On the base of this finding, associated to histopathological features and the clinical date, a diagnosis of subcorneal pustular dermatitis (SCPD, Sneddon-Wilkinson disease) was made.

**Figure 4:**
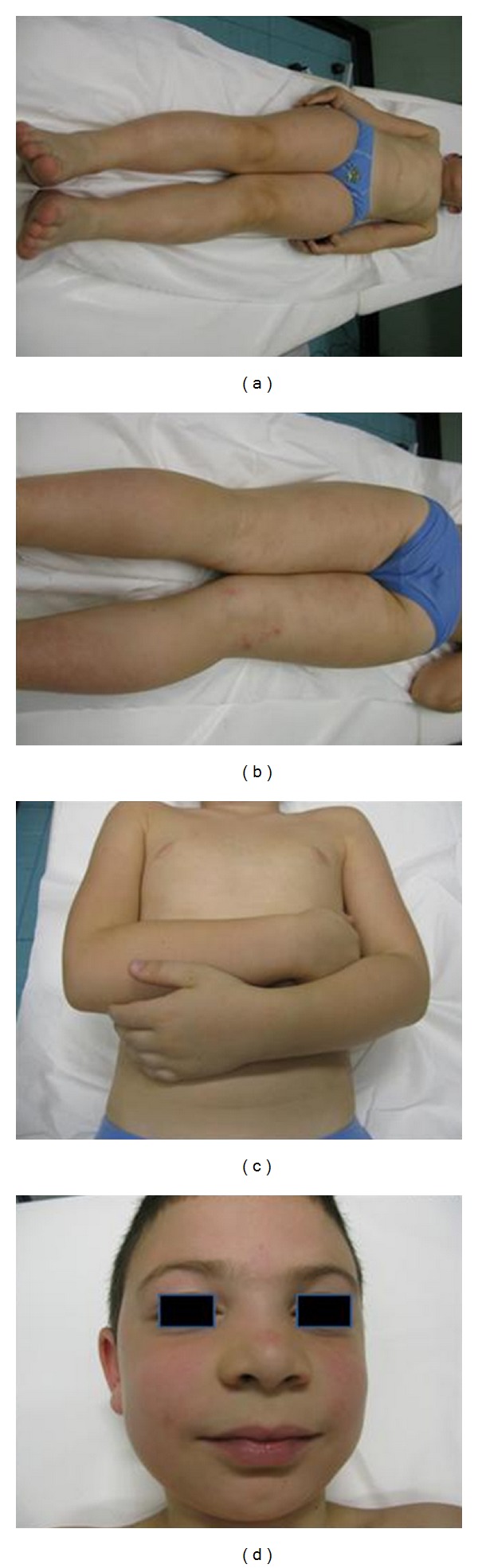
Followup at 2 weeks. The patient was treated with Dapsone. The cutaneous lesions were almost completely healed.

The patient was treated with 30 mg of oral Diaminodiphenylsulfone (Dapsone, 1 mg/kg/day). The cutaneous lesions were almost completely healed at the first followup, within 2 weeks (Figure 4). After 4 weeks, treatment with Dapsone was continued on alternate days for another month, at the same daily dosage.

After 3 months, the patient is still monitored for the followup at our hospital every 2 weeks.

## 3. Discussion and Review

Children can have various bullous and pustular skin diseases like pemphigus vulgaris, pemphigus foliaceus, bullous pemphigoid, pustular bacterid, and psoriasis as well as dermatitis herpetiformis [8, 9]*;* all of these were once thought to be unique to people in the fourth-fifth decade of life. Subcorneal pustular dermatosis appears to be another one of these diseases.

The etiology of the disease is still obscure. There are well-documented SCPD in associations with benign monoclonal IgA gammopathy [10] and pyoderma gangrenosum [11]. There are also reports in association with IgA myeloma [3], SAPHO syndrome [12], Crohn's disease [13], Sjogren's syndrome [14], rheumatoid arthritis [15], and thyroidal diseases [16].

In our case, the history, physical examination, and laboratory results did not reveal any systemic associations. Moreover some cases, which were consistent with SCPD according to the clinical and histologic features, have been reported with the presence of an intercellular IgA deposition within the epidermis [17]. 

This disease involves more frequently the trunk, intertriginous areas, and flexor aspects of the limbs; more rarely the face is implicated, as in this case. Pustules on palms and soles have also been reported [18], while mucous membranes are almost never affected.

The differential diagnosis of SCPD includes pustular psoriasis, impetigo, dermatophyte infection, and immunobullous diseases (dermatitis herpetiformis, pemphigus, linear IgA disease, and intercellular IgA diseases). Unlike pustular psoriasis, nails and scalp are uncommonly affected in SCPD; moreover spongiform pustules, formation of microabscess, and elongation of rete ridges do not occur in classical Sneddon-Wilkinson disease [17]. In generalized pustular psoriasis, patient cases to have fever and leukocytosis [19]. A dermatophyte infection can be easily excluded with a direct microscopic examination of fungal elements. The differential diagnosis from impetigo may be difficult; the possible bacterial contamination, not always, can be demonstrated by Gram stain. IgA deposition in the dermal papillae distinguishes SCPD from dermatitis herpetiformis.

In the IgA pemphigus subtype, generally, the acantholysis tends to be more pronounced than in SCPD, Sneddon-Wilkinson disease, in this regard, in our sample, were not observed acantholytic cells; moreover, in the IgA pemphigus, DIF studies demonstrate intercellular IgA accumulation in squamous cells.

In Sneddon and Wilkinson's original report [1], the average age of the patients was 54.8 years.

Only 15 cases of pediatric SCPD are described in literature [2, 20–29].

Sarkany [20] had a patient 10 years of age and Beck et al. [21] had had a 29-years-old patient whose disease had started when the patient was 3 months old. Baker and Ryan [22, 23], in their two reviews of 104 cases and 155 cases of pustular psoriasis, reported that generalized pustular psoriasis had its onset before the age of 11 in only four children (three boys and one girl). All of the four children had the “annular pattern” of pustular psoriasis that according to the authors, somewhat resembled subcorneal pustular dermatosis.

R. E. Burns, MD, had a patient with subcorneal pustular dermatosis who gave birth to a child with similar skin lesions that lasted seven days (oral communication, April 1973) [2].

Desmons and Defrenne [24] also described in 1973 a case of a 12-year-old boy with a recurrent subcorneal pustular dermatosis.

Johnson and Cripps [2] report two 3-year-old children, a boy and girl, with Sneddon-Wilkinson syndrome; in both of these children some of the flares of the disease followed infections. 

Garg et al. [25] reported in 1985 a case of a young boy affected by SCPD successfully treated with dapsone. 

In 1986 Rosińska-Borkowska and Henig published a case report about a 30-month-old patient with SCPD [26]; in the same year Park et al. described a 12-year-old boy with a 5-year history of recurrent generalized dermatoses with scales, crusts, and pustole: Dapsone and prednisolone with a topical fluocinolone acetonide did not produce improvement [27]. The skin lesions cleared completely after 11 exposures of UV-B three times a week. After 8 months of followup, there has been no recurrence.

In 2003 Koçak et al. [28] had a 13-year-old girl with SCPD in terapy with Dapsone. After 3 weeks of treatment, the lesions regressed almost completely, but her hemoglobin decreased from 12.5 mg/dL to 9.6 mg/dL; for this reason systemic treatment was stopped and topical steroid ointment was initiated with acceptable clinical results.

The last case reported in literature shows a case of juvenile subcorneal pustular dermatosis successfully treated with acitretin [29].

Even if SCPD is an uncommon condition in childhood, it must be considered as a possible cause of sterile pustular eruptions in a child. An accurate physical examination, a complete blood count, and studies of serum biochemistry are strongly recommended to exclude a pathology in association. Dapsone remains the treatment of choice but its safe is still debatable and a close followup is required.
